# Psychological health and wellbeing of primary healthcare workers during COVID-19 pandemic in Malaysia: a longitudinal qualitative study

**DOI:** 10.1186/s12875-022-01870-0

**Published:** 2022-10-13

**Authors:** Ee Ming Khoo, Adina Abdullah, Su May Liew, Norita Hussein, Nik Sherina Hanafi, Ping Yein Lee, Khatijah Lim Abdullah, Lelamekala Vengidasan, Ahmad Ihsan Bin Abu Bakar, Hilary Pinnock, Tracy Jackson

**Affiliations:** 1 Department of Primary Care Medicine, Faculty of Medicine, Universiti Malaya, Kuala Lumpur, Malaysia; 2 Faculty of Medicine, UMeHealth Unit, Universiti Malaya, Kuala Lumpur, Malaysia; 3 Department of Nursing School of Medical and Life Sciences, Sunway University, Subang Jaya, Malaysia; 4 NIHR Global Health Research Unit On Respiratory Health, University of Edinburgh, Edinburgh, UK

**Keywords:** COVID-19, Healthcare workers, Longitudinal qualitative study, Primary care, Psychological impact

## Abstract

**Background:**

Primary healthcare workers (PHCWs) are at the frontline of dealing with viral pandemics. They may experience significant psychological stresses, which have hitherto not been examined in depth. We aimed to explore the impact of the COVID-19 pandemic on the psychological health and wellbeing of frontline PHCWs in Malaysia.

**Method:**

We purposively recruited PHCWs with diverse backgrounds in Klang Valley, Malaysia. Using longitudinal qualitative methods, we conducted two sequential semi-structured telephone interviews, 3 to 4 weeks apart, to capture different stages of the pandemic. Interviews were audio-recorded, transcribed verbatim, and analysed thematically.

**Result:**

Twenty-one PHCWs participated yielding a total of forty-two interviews. Themes clustered around stressors associated with work, home, and leisure activities, emotional changes, and modifying factors. In the first interviews, COVID-19 had just started in Malaysia. Participants expressed fear about the actual and perceived personal risk of COVID-19 infection. Most were worried about transmitting COVID-19 to their family members. Some felt stigmatized because of this perceived risk of infection. By the second interviews, participants felt safer, but instead focused on the need to keep other people safe. Participants’ emotions were influenced by their perceived risk of contracting COVID-19 infection. Internal factors such as religion enabled them to manage their concerns and develop personal coping strategies. Support from family members, colleagues, and employers promoted wellbeing during the pandemic. Training sessions, daily roll calls, and psychological support services were important in maintaining their psychological health and wellbeing. Many participants were hopeful and believed normalcy would return by the end of 2020.

**Conclusion:**

PHCW’s psychological health and wellbeing evolved throughout the early stages of the pandemic and were influenced by their perceived risk of contracting the disease and personal belief structures. Clear updates on the disease and strategies for keeping safe at work and socially are essential to maintaining PHCWs’ psychological health and wellbeing.

**Supplementary Information:**

The online version contains supplementary material available at 10.1186/s12875-022-01870-0.

## Introduction

Viral pandemics are not new phenomena. The 1918 Influenza A H1N1 pandemic killed 50 million people worldwide [1]. Since then, several other viral pandemics have occurred, including the Human Immunodeficiency Virus (HIV) beginning in 1981, the Severe Acute Respiratory Syndrome Coronavirus (SARS-CoV) in 2002, the Influenza A virus subtype H1N1 (A/H1N1) in 2009, the Middle East Respiratory Syndrome Coronavirus (MERS-CoV) in 2012, and the Ebola virus in 2013 [2]. The first human case of COVID-19 was identified in December 2019 in Wuhan, China, with flu-like symptoms and pneumonia [3].

In March 2020, the World Health Organization (WHO) declared COVID-19 a pandemic. Since then, it has spread rapidly across all continents, affecting nearly every country on the planet, leading to 499 million positive cases and 6.18 million deaths worldwide as of April 13, 2022 [4]. Malaysia had 4.34 million COVID-19 positive cases, and 35,341 deaths up until April 2022. The Malaysian government enacted a Movement Control Order (MCO), equivalent to a total lockdown, on 18 March 2020 to break the COVID-19 chain [5], followed by a Conditional MCO on 4 May 2020 [6] and a Recovery MCO, on June 10, 2020, when COVID-19 incident cases had reduced to double digits [7].

Primary healthcare workers (PHCWs) are on the frontline of the fight against the pandemic. Several studies have found that the COVID-19 pandemic has affected the mental health and wellbeing of healthcare workers [8–10], who have experienced anxiety [8], fear, depression [9], insomnia, and stress [10]. The COVID-19 pandemic had been shown to have a moderate to severe psychological impact on frontline healthcare workers and the effects were different for various groups [11, 12]. Those with a higher likelihood of direct COVID-19 exposure were found to be more likely to suffer from mental health issues [13, 14]. Despite increased prevalence of psychological issues among healthcare workers during the pandemic, few studies have explored the reasons for these observations. We aimed to explore how the COVID-19 pandemic affected the psychological health and wellbeing of frontline primary healthcare workers during the early phases of the pandemic.

## Methods

In this longitudinal qualitative study, two sequential interviews were performed with each participant to explore their psychological health and wellbeing during the pandemic. Longitudinal qualitative research is highly sensitive to contextual issues and can shed light on important micro-social processes, such as how people subjectively negotiate the changes that occur in their lives during times of personal life transition [15]. A narrative research constructivist approach was used to answer the study objectives. The first interviews were undertaken in the early pandemic period (June/July 2020) during the first lockdown known as a Movement Control Order (MCO) in Malaysia and the second interviews approximately a month later (July/August 2020) during the Recovery MCO when COVID-19 cases had reduced from double to single digit and restrictions were relaxed, interstate travel was permitted, and schools were reopened. PHCWs working at the frontline in Klang Valley, Malaysia (Kuala Lumpur, the capital city and adjacent state), were interviewed. All researchers were experienced in qualitative research. Ethical approval was obtained from the Medical Research Ethics Committee, University Malaya Medical Centre (202,058–8605), and The Academic and Clinical Central Office for Research and Development, The University of Edinburgh (AC20056) sponsored the study**.** All methods were carried out in accordance with relevant guidelines and regulations.

### Participants

We purposively recruited a diverse group of about 20 professionals with different demographic profiles (age and gender), place of work (private or public), and professional roles (clinic administrative staff, postgraduate Family Medicine trainees, general practitioner (GP), Clinic nurse) for maximum variation.

### Participant recruitment

All public PHCWs were recruited from a Primary Care Clinic in a teaching hospital. We used the clinic staff name list and purposively sampled staff according to different groups. We approached them individually for consent. As for the private PHCWs, they were recruited through the snowballing method. Potential participants were contacted through a telephone call, after which the PIS dan consent forms were emailed to them for their consideration. A written informed consent was obtained from all subjects and/or their legal guardian(s) before the start of the initial interview and confirmed verbally before the second interview. All methods were carried out in accordance with relevant guidelines and regulations.

We chose this sample size as being sufficient to allow us to hear a range of perspectives, and realistic within the funding envelope, though we had some flexibility according to whether we had achieved data saturation in the initial interviews. Sample sizes were determined by data saturation [16].

### Interviews

Two semi-structured interviews were carried out with each participant between 2 June and 28 Aug 2020 in their preferred language, Malay (national language) or English. The interviews were audio-recorded, transcribed verbatim and the Malay transcripts were translated to English, prior to thematic analysis, using a forward translation method by researchers fluent in both English and Malay languages.

A topic guide (Additional File 1), developed based on the Health Belief Model [17] and reviewed by a multidisciplinary research team, was used to guide the telephone interviews. Participants were asked about their background, nature of their job, their role as a COVID-19 frontline worker, their emotional state, and psychological impact of the pandemic. Questions were open-ended to allow the participant to control the level of detail they wanted to share about their experience. Three researchers (two academic GP, and one academic nurse practitioner) who are experienced qualitative researchers conducted the interviews. Before starting the interview process, all three researchers went through the topic guide and discussed ways to standardise the way the questions being asked. Each participant was interviewed by one of these researchers and the same researcher conducted the follow-up interview a month later to revisit themes from the first interview and explore any changes in the participant’s psychological status and wellbeing. At the end of the second interview, the interviewer wrote a reflective note of any changes in the participant's feelings and views over the two interviews. Data were saturated after 18 participants and three more participants were interviewed to ensure no new codes emerged. All participants were offered the opportunity to review their transcript, but all declined.

### Data analysis

Analysis was facilitated by a qualitative data software program (QSR NVivo version 12; QRS International, Doncaster, Australia). All researchers and one trained research assistant independently coded paired transcripts for one participant using a thematic narrative approach [18] and consensus was reached on the coding frame. Thematic analysis (TA) is a method for identifying, analyzing, and interpreting patterns of meaning (‘themes’) within qualitative data [19]. The rest of the paired transcripts were coded by main author and a research assistant using this frame. Codes were grouped to form themes, which were discussed and agreed with the research team to reduce subjectivity of findings.

### Data availability

The datasets generated and/or analysed during the current study are available in the DataVault repository, https://datavault.ed.ac.uk

### Reflexivity

Reflexivity refers to being aware of how the researcher and the research process can influence the data collected [20]. All three researchers are all experienced qualitative researchers, who constantly reflected on how their interactions with participants might be influenced by their own professional background, experiences, and prior assumptions. The participants were interviewed by researchers who were not known to them, and this had facilitated them to talk openly about their experiences and feelings. The team of investigators included eight GPs, one psychologist, and one academic nurse. We conducted a number of multidisciplinary meetings to discuss the findings and came to a consensus.

## Results

A total of 21 participants were recruited out of 25 approached (four clinic administrative staff, five postgraduate family medicine trainees, four nurses, five general practitioners, and three GP clinical assistants) and 42 interviews were recorded. Participant characteristics are listed in Table 1. The interval between the first and second interviews ranged from three to four weeks. Each interview lasted on average 30 to 35 min.

**Table 1:** Sociodemographic profile of participants

**Characteristics**		**Numbers (*n* = 21)**
Age (years)	25–35	8
	36–45	7
	46–55	6
Sex	Female	16
	Male	5
Marital status	Single/Divorced	5
	Married	16
Ethnicity	Malay	14
	Chinese	3
	Indian	4
Occupation	Clinic Administrative Staff	4
	Postgraduate Family Medicine trainee	5
	General Practitioner (GP)	5
	GP Clinical Assistant	3
	Clinic Nurse	4
Healthcare sector	Public	16
	Private	5

### Overview of themes

**Fig. 1:**
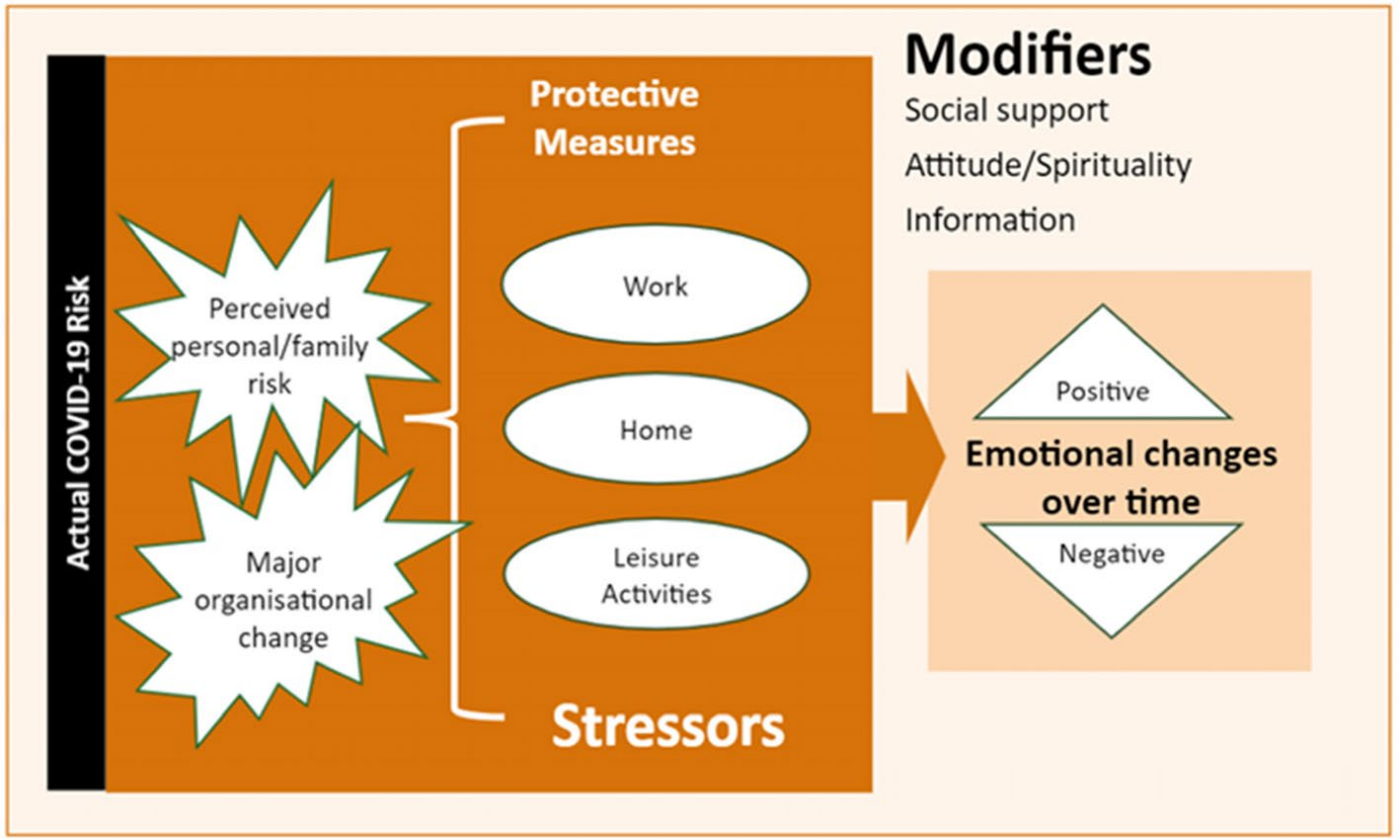
Relations between stressors, risks (actual/perceived), emotion, and modifiers

Three themes emerged from the analysis: stressors associated with work, home, and leisure activities; emotional changes; and modifying factors. Figure 1 presents the themes.

### Theme 1: Stressors associated with work, home, and leisure activities

Participants were stressed by the changes to their daily routines and habits due to the MCOs and healthcare system responses to the pandemic. These changes occurred at their workplace influencing their clinical practice as well as at home affecting their lifestyle.

*“About working during the pandemic, … there is no pressure working during the pandemic. It’s just that you have to be really careful. A lot said to wash hands and all, just follow the right steps, how to wear the PPE (Personal Protective Equipment), methods of wearing, proper disposal, and all, right. We have to do all correctly.”*

Participants, particularly those working in the private settings noted a reduction in the number of patients attending their clinics, so that they were worried about their income.

*“The change occurred because when COVID happened, our clinic received lukewarm support. Meaning there were not many patients coming in. And sometimes we did not receive any patients at all.”*

P18, 1^st^ interview, age 25–35 years, GPclinical Assistant, Private

At home, fear of transmitting the disease to family members led some participants to adopt precautionary measures such as taking a bath immediately after coming home from work and washing their clothes separately from other family members' clothes. One participant shared how frustrated and isolated they felt because as a healthcare worker, she was viewed as having a very high risk of contracting COVID and was not accepted even by her own family members.

*“Aa so when we reach home, we would shower immediately before we meet our children regardless if they are crying or not, we take a shower first, wash everything, separate all the clothes, wash it first, only after all that we can meet our children.”*

P1, 1^st^ interview, age 36–45 years, Clinic administrative staff, Public

*“And er, and it made my life a little bit difficult because when I ask my, er ask people to help, find people to take care of my kids and my mother, my own mother will say, ‘You think people want to take care (of your children)? they know you are a doctor you know, very high risk they don’t want to take care.’ You know they, they will give this kind of comments …”*

P12, 1^st^ interview, age 36–45 years, Postgraduate family medicine trainee, Public

Some experienced disruptions in their leisure activities, their lifestyles changed from leading an active lifestyle to a more sedentary one.

*“Ha, I don’t go jogging, I don’t go shopping. I don’t do anything at all. I just go outside, to Giant [supermarket], buy groceries, and come straight back home. Really none. I used to really go for facial and massage every month.”*

P8, 1^st^ interview, age 46–55 years, Clinicnurse, Public

“It changed from physical to more sedentary, where you just sit and watch movies, play with your phone.”

P11, 1^st^ interview, age 36–45 years, Postgraduate family medicine trainee, Public

### Theme 2: Emotional changes

Participants were expressing sadness, fear, and feeling scared when talking about their emotions. There was fear of exposure to COVID-19 as working in healthcare settings and of spreading the infection to family members, some were vulnerable.

*“Yes, that is what I said, always pray, dear Allah if I held the personal identification card and I used sanitiser hopefully there is nothing, may my family stay healthy, and that I have no infection, because there is also elderly in my family.”*

P2, 2^nd^ interview, age 46–55 years, Clinic administrative staff, Public

*“I live with my parents and my husband who works in a hospital. So, we don’t want to pass it to my mom, we don’t want my mom to pass it to my grandmother, then my mom would be considered as ‘you’re being exposed too’ …. a lot of fear there.”*

P6, 1^st^ interview, age 36–45 years, General Practitioner, Private

Participants were unable to visit their family and friends due to the MCOs. As a result, they had negative feelings. The distress of being unable to visit their loved ones was shared by most participants.

*“The restriction of movement, that largely affected me because I cannot go home as usual. Cannot meet my kids. Because I normally go back every weekend to see my kids and my husband in Kedah (another state).”*

P11, 1^st^ interview, age 36–45 years, Postgraduate family medicine trainee, Public

“Negative thing was not able to meet your loved ones and family members during that MCO period. And on top off it is the burden to serve.”

P16, 1^st^ interview, age 25–35 years, Postgraduate family medicine trainee, Public

A few participants were very stressed at home, experienced increased tension in interactions with family members, and started having arguments with their partners. Some were very disappointed with their spouse or family members who they felt did not support them emotionally or physically. Some were worried about the children’s education and future.

“I did go to the stores once in a while, but yes, I think we were threading on eggshells in the first month. The smallest things would cause us close to just having a breakup.”

P11, 1st interview, age 36–45 years, Postgraduate family medicine trainee, Public

“But in terms of the future and our children’s education there is still some uncertainty, so it has gotten better. In terms of uncertainties, it does cause worry.”

P11, 1^st^ interview, age 36–45 years, Postgraduate family medicine trainee, Public

Although most emotions expressed during the first interviews were negative, there were some positives. Several participants finally had some time to start a hobby like gardening, embroidery and watching their favourite movie or series and do online shopping.

“Er I did er, find some time to do things that I like. It’s that it releases my stress. So, what I like to do is gardening. So, I, I planted vegetables.”

P12, 1^st^ interview, age 36–45 years, Postgraduate family medicine trainee, Public

“Actually I, I take up embroidery and, and cross stitching.”

P20, 1^st^ interview, age 25–35 years, Postgraduate family medicine trainee, Public

Some participants in the second interviews had found precious time for self-reflection and soul searching.

“Make sure that we take care…take care of ourselves. Haha…take care of ourselves.”

P12, 2^nd^ interview, age 36–45 years, Postgraduate family medicine trainee, Public

“But I think enough self-reflection. (chuckles) Just to do everything you want to do in life (laughs).”

P20, 2^nd^ interview, age 25–35 years, Postgraduate family medicine trainee, Public

Participants felt safe and assured by the factual updates of the Director General of Health’s daily media conference in the national channel. They expressed confidence in the Director General of Health’s handling of the pandemic.

*“I only listen to his [Director General of Health’s] announcements and that’s it. I don’t listen to anyone else (laughs). Um… because he just gives the… He gives out facts and… and whatever the things to do… the best actions at that time with whatever… studies or um… what’s the word here… data that they have from previous pandemic..”*

P15, 1^st^ interview, age 46–55 years, General Practitioner, Private

### Theme 3: Modifying factors

Psychosocial modifying factors such as work and social support, attitude or spirituality, and information played a crucial role in the participants’ ability to cope with the pandemic. Social support from colleagues, family members, and friends was a pillar of guidance and encouragement.

“For the time being, yes. I think because I have quite a good support system.”

P7, 2^nd^ interview, age 25–35 years, General Practitioner, Private

*“Yeah, I do receive support… Because I have a meeting every day, so they kind of asking how everyone is doing, so updating each other whether we are well, and some of my superiors as well, they kind of know my situation, so they kind of give personal or support to me, giving advice.”*

P11, 1^st^ interview, age 36–45 years, Postgraduate family medicine trainee, Public

Physical and emotional support from family such as help with childcare and house chores and sharing motivational and positive words could provide relief to stressed PHCWs.

*“Husband. Sometimes when I’m too tired of working, “it’s okay sleep” he said. He will take care of the children. He supported me a lot. Even my in-laws would say “it’s okay let me cook”.”*

P4, 1^st^ interview, age 25–35 years, Clinic administrative staff, Public

*“Actually the, the, the person that really supports me is my wife. Er she has been supportive. But I would say my parents, in-law, not, they don’t understand what we are going through because they are not in the healthcare frontline, I can say that.”*

P12, 1^st^ interview, age 36–45 years, Postgraduate family medicine trainee, Public

Internal factors, like spirituality and attitude, enabled participants to develop personal coping strategies. Some of them believed this was a test from God, and praying provided comfort during this challenging time.

*“Hmm… the risk is still there, but I just leave it to God actually. If God says I should be well, actually it’s all written in the qada’ and qadr, [your destiny] it’s just that, as a prevention I have to…”*

P18, 2^nd^ interview, age 46–55 years, Clinic nurse, Public

“If from a Muslim’s point view, Allah wants to give us this test okay, we have to be strong, accept, aa so we have to fight for it.”

P19, 1^st^ interview, age 25–35 years, GP clinical Assistant, Private

Participants also agreed that a consistent source of trustable information lessened their nervousness and anxiety. Several participants suggested having training sessions, daily roll call sessions, and a formal support center to improve their mental health and wellbeing.

*“Because information is always around, right? It was through Facebook, then through roll call. Everyday have roll call, always reminding us. That thing, when we always be reminded of, it’s already set in our minds that it is important.”*

P4, 1^st^ interview, age 25–35 years, Clinic administrative staff, Public

*“First, I suggest for the rollcall to be done if possible. Okay. I mean doctors delivering talks for us, short CME [Continuing Medical Education]. Just for a while, it’ll be okay, I guess. It could be about anything. As long as I have the knowledge.”*

P8, 1^st^ interview, age 46–55 years, Clinic nurse, Public

### Evolving perceptions

By the time of the second interview, participants felt more positive. Some noted that patients had adapted to the new norm as they wore masks and made appointments before attending clinics. Their workload had also returned to normal.

*“They are still the same, I would say. But like, for example, patients who come here, I think they still, they are quite alright. They still wear their masks, and they follow the instructions. In the public mainly now, more people are travelling.”*

P14, 2^nd^ interview, age 25–35 years, Postgraduate family medicine trainee, Public

*“Yep, there are many patients again. But we still go by appointment. Meaning they cannot come in big numbers altogether. Haa. We will limit each person. So every day we will limit how many people we can accept, and how many people can wait in the clinic, as well as outside the clinic.”*

P18, 2^nd^ interview, age 25–35 years, GPclinical Assistant, Private

*“Er in terms of job scope I think it’s about the same as er of the last time we talked. But in terms of the attendance of patients they are definitely coming back, we, I think we can say that it back to like being before the COVID started.”*

P20, 2^nd^ interview, age 25–35 years, Postgraduate family medicine trainee, Public

However, there were patients who had not adapted to the “new normal” and frontline staff had to enforce the standard operating procedures. Participants were clear on the safety measures that they needed to reinforce.

*“It’s more challenging. We have to be patient. Like, since the morning, we will be screaming at the patients (in the clinic). We have to tell them the procedure that needs to be done, have they done the entry visa (declaration of symptoms) or not.”*

P9, 2^nd^ interview, age 25–35 years, Clinic nurse, Public

After the re-opening of social and economic activities, family members became more relaxed about mixing with frontline healthcare workers. However, around the time of the second interview, schools were beginning to open and some participants were anxious about their children contracting COVID-19 at school. One participant with school-age children decided not to send her children back to school.

“Ya, it’s getting better. They, they are more receptive la. So, like, like tonight, tonight I’m going to have dinner at my in-laws’ place.”

P12, 2^nd^ interview, age 36–45 years, Postgraduate family medicine trainee, Public

*“Because one thing, we are scared, they (our children) might get infected. Another thing to think about their studies also. So, I am undecided (whether to send my children to school). I still a bit worried. But if my kids have any symptoms, then I think I won’t send them la. Scared (they) will spread to others.”*

P13, 2^nd^ interview, age 36–45 years, Clinic nurse, Public

During the second interviews, participants also shared positive feelings with regards to their leisure activities. They continued to practice infection control precautions and felt that the economy was returning to normal. Some had spent more time with their families and were interacting socially with their neighbours.

*“I can go exercise and bring (my) kid to the playground. Maybe have more social interaction with the neighbor. And I... quite often I can go home, go back to my parents' home, parents' house and visit them.”*

P5, 2^nd^ interview, age 36–45 years, General Practitioner, Private

Most of the participants felt much safer by the time of the second interview and were optimistic about going back to normality by the end of the year 2020.

## Discussion

### Summary of findings

Frontline PHCWs were fearful of the actual and perceived personal risk of getting COVID-19. Most were worried about transmitting the disease to their family members and some feared contracting COVID-19 themselves due to their high-risk job and their ‘duty to serve’ where they felt personal responsibilities to be at work as frontline healthcare workers. The changes at their workplace such as the new procedures to prevent or de-risk exposure to positive COVID-19 patients also influenced their mental health. Some PHCWs felt stressed with the added protocols and practices. In the private sector, there was worry about lost income. Some participants reported discrimination or social stigma as healthcare professionals were deemed to be at high risk of infection. Despite this, some participants had positive emotions such as having more time with their families during lockdown and had started new hobbies. Summaries of the differences in the themes between the two interviews are presented in Table 2.

**Table 2:** Summaries of differences between 1st and 2nd interview codes

Themes	1^st^ Interview	2^nd^ Interview
Stress and changes		
• Work	Reduced patient visits in the private sector	Workload back to normal Risk reduced when patients practiced standard operating procedures (SOPs) although some patients still needed to be reminded
• Home	Fear of transmitting the virus to family and friends The stigma of being a PHCW	More acceptance of PHCWs within the social circle Other activities were opening eg: school
• Leisure activities	Unable to go for a jog Switched to sedentary activity	Continue to observe SOPs Positive feeling about economy Usual activity was returning to normal
Emotional changes		
• Positive	Starting a new hobby and having more time for themselves Felt reassured with factual information relayed in daily press conference given by the Ministry of Health	Visited parents at hometown and more social interaction with neighbors Went for a holiday Family more receptive, less stressed about risk
• Negative	Stressed and sad because of movement restriction order, unable to meet a loved one Worried about infection risk or family members who were vulnerable	Fear of children getting COVID-19 as school reopened
Modifying factors		
• Social support	Supportive working colleagues Challenges at home from unsupportive family members	Could identify more support at home and at work
• Attitude/ Spirituality	Trust in God with the hope that he would ease everything	Felt content and happy Confident with the government and workplace leader
• Information	The need for more information Medical knowledge and training were sources of confidence	Information provided in a regular roll call was useful Relied only on trusted sources

### Strength and limitations

The main strength of this study is that we sampled frontline healthcare workers from varied occupational backgrounds including medical and non-medical personnel, as well as those working in public and private sectors. The longitudinal qualitative study design enabled us to capture the effect of external variables such as the changing restrictions and numbers of positive COVID-19 patients on PHCWs’ psychological health and wellbeing. We were able to capture the temporal and situational impact on the participants' psychological state as evidenced by the quotes reported in the result sections.

The main limitation of our study is that the 21 frontline PHCWs in our study may not be representative of all frontline PHCWs and we may have different results from a different group of participants. We also observed that domestic status and caring responsibilities had a significant impact on participants’ experiences. We could not report on it as one of the demographic characteristics of participants because we did not collect it prior to the interviews, and we did not use it as a variable in our purposive sampling. Therefore, our findings may not represent all contexts. Also, the workplace environment may have contributed to the findings and only be applicable to the public and private healthcare clinics that were involved in the study. At the time of writing, the COVID-19 pandemic has been ongoing for over two years and the findings from this study only reflect the impact it had at the early stages of the pandemic.

### Discussion in relation to published literature

We found the main factor contributing to stress amongst healthcare workers during the pandemic was their perceived risk of contracting—or passing on—infection. Juggling their commitment to the job, the risk of infection, and the risk of transmitting it to their family faced healthcare workers with critical and occasionally impossible decisions which resulted in extreme stress. Self-perceived infection vulnerability and transmission to family had emerged as predictors of poor mental health among HCWs in previous studies [21, 22]. Fear of infectivity in HCWs can also be a result of poor health system preparedness, such as a lack of personal protective equipment (PPE) and adequate training during a pandemic [23–25], which were not identified as major issues among our participants.

PHCWs and their family members reported stigma from their own family and society as they were believed to be at high risk of contracting COVID-19. High percentages of health care workers also reported social stigmatisation during the 2003 SARS outbreak in Singapore and the Ebola epidemic in Sierra Leone [26, 27]. Our findings are consistent with previous research [28, 29] that social stigmatization during the COVID-19 pandemic has a significant impact on the psychological state of HCWs. To address such stigma during a pandemic, it is important for family members and society to understand about viral transmission and health and safety practices implemented in healthcare settings to reduce the risk of infection among HCWs, such as the use of personal protective equipment. Greater awareness and understanding could reduce stigma on PHCWs’ and improve acceptance and increase support that is much needed from PHCWs family and friends [30].

PHCWs in the private sector faced different challenges during this pandemic. The lockdown contributed to major loss of income in the private healthcare system especially in developing countries [31, 32]. Fewer patients presented themselves to the private primary care clinic, preferring to be treated at the public healthcare centers. Reports of positive COVID-19 patients visiting a private clinic had a devastating effect on patient numbers [33]. On the other hand, PHCWs in the public sector had to deal with changes in the work process and implementation of new SOPs which in turn increased the burden on frontline PHCWs. The psychological impact of the COVID-19 pandemic had been shown to be a result of economic loss, during the pandemic [34]. A recent paper exploring the impact of switching to remote consulting during the COVID-19 pandemic on general practitioners (GPs) found that GPs felt high volumes of remote consultations to be more mentally intense and less satisfying. Some felt an increased strain in making clinical decisions, prescribing, and holding more clinical risk when delivering telephone consultations [35].

Despite the stress, some PHCWs expressed positive emotions as they had more time for themselves, family, and friends. This has been similarly observed in other regions, where lockdown brought increased work and family satisfaction [36–38]. However, lockdown also created an extraordinary burden for working parents [39, 40] as schools were shut and some participants had to care for them at home and supervise their home schooling.

### Implications

This study highlights the need for psychological support from the beginning of a pandemic to support PHCWs’ mental health and wellbeing. Some countries, like China [41] and Pakistan [25], have implemented psychological support groups run by professionals like psychologists and psychiatrists with some success. This study also highlighted that brief informal support provided by colleagues at work (such as a short morning roll call) is enough to make PHCWs feel supported. A clear plan also needs to be in place to help them cope with issues outside the workplace that may add to their stress and affect their emotions. Similar study should be performed at later stages of the pandemic to fully explore how PHCWs psychological health is impacted.

## Conclusions

The COVID-19 pandemic has had an unprecedented impact on the Malaysian PHCWs. They faced numerous challenges since the beginning of the pandemic, including learning new organisational procedures, dealing with positive COVID-19 patients, and managing changes that occurred in their professional and personal lives. This affected their psychological health and wellbeing. Frontline PHCWs need support not only from their family and the healthcare systems but from all government sectors and society to tackle their worries and fears. 

## Supplementary Information

**Additional file 1:** 

## Data Availability

The datasets used and/or analysed during the current study are available from the corresponding author on reasonable request.

## References

[1] Taubenberger JK, Kash JC (2011). Insights on influenza pathogenesis from the grave. Virus Res.

[2] Roychoudhury S, Das A, Sengupta P, Dutta S, Roychoudhury S, Choudhury AP, Ahmed ABF, Bhattacharjee S, Slama P (2020). Viral pandemics of the last four decades: pathophysiology, health impacts and perspectives. Int J Environ Res Public Health.

[3] Liu YC, Kuo RL, Shih SR (2020). COVID-19: The first documented coronavirus pandemic in history. Biomed J.

[4] WHO Coronavirus (COVID-19) Dashboard

[5] Coronavirus: Malaysia in partial lockdown from March 18 to limit outbreak [https://www.scmp.com/week-asia/health-environment/article/3075456/coronavirus-malaysias-prime-minister-muhyiddin-yassin]

[6] CMCO gets mixed reactions from state governments [https://www.bernama.com/en/news.php?id=1838131]

[7] CMCO ends June 9, Recovery MCO from June 10 to Aug 31 (Updated) [https://www.thesundaily.my/home/cmco-ends-june-9-recovery-mco-from-june-10-to-aug-31-updated-EM2538754]

[8] Temsah MH, Al-Sohime F, Alamro N, Al-Eyadhy A, Al-Hasan K, Jamal A, Al-Maglouth I, Aljamaan F, Al Amri M, Barry M (2020). The psychological impact of COVID-19 pandemic on health care workers in a MERS-CoV endemic country. J Infect Public Health.

[9] Teo WZY, Yap ES, Yip C, Ong L, Lee CT (2021). The psychological impact of COVID-19 on 'hidden' frontline healthcare workers. Int J Soc Psychiatry.

[10] Lin K, Yang BX, Luo D, Liu Q, Ma S, Huang R, Lu W, Majeed A, Lee Y, Lui LMW (2020). The mental health effects of COVID-19 on health care providers in China. Am J Psychiatry.

[11] Elbay RY, Kurtulmus A, Arpacıoğlu S, Karadere E (2020). Depression, anxiety, stress levels of physicians and associated factors in Covid-19 pandemics. Psychiatry Res.

[12] Que J, Shi L, Deng J, Liu J, Zhang L, Wu S, Gong Y, Huang W, Yuan K, Yan W (2020). Psychological impact of the COVID-19 pandemic on healthcare workers: a cross-sectional study in China. General Psychiatry.

[13] Wang L-q, Zhang M, Liu G, Nan S, Li T, Xu L, Xue Y, Zhang M, Wang L, Qu Y: Psychological impact of coronavirus disease,  (2019). (COVID-19) epidemic on medical staff in different posts in China: a multicenter study. J Psychiatr Res.

[14] Ferreira EAL, Valete COS, Santos A, Passarini JNS, Silva AE, Miwa MU (2021). Health care professionals and end-of-life care during the COVID-19 pandemic. Rev Assoc Med Bras (1992).

[15] Holland J, Thomson R, Henderson S: Qualitative longitudinal research: a discussion paper. 2006.

[16] Bryman A: How many qualitative interviews is enough. 2012(How many qualitative interviews is enough):18–20.

[17] Glanz KRBKVK: Health behavior and health education : theory, research, and practice. San Francisco, CA: Jossey-Bass; 2008.

[18] Hargood C, Millard DE, Weal MJ: A thematic approach to emerging narrative structure. In: Proceedings of the hypertext 2008 workshop on Collaboration and collective intelligence. Pittsburgh, PA, USA: Association for Computing Machinery; 2008: 41–45.

[19] Braun V, Clarke V: Thematic analysis. In., edn.; 2012: 57–71.

[20] Haynes K: Qualitative Organizational Research: Core Methods and Current Challenges. In. 55 City Road 55 City Road, London: SAGE Publications, Inc.; 2012.

[21] Golechha M, Bohra T, Patel M, Khetrapal S (2022). Healthcare worker resilience during the COVID-19 pandemic: a qualitative study of primary care providers in India. World Med Health Policy.

[22] Hong S, Ai M, Xu X, Wang W, Chen J, Zhang Q, Wang L, Kuang L (2021). Immediate psychological impact on nurses working at 42 government-designated hospitals during COVID-19 outbreak in China: a cross-sectional study. Nurs Outlook.

[23] Kaye AD, Okeagu CN, Pham AD, Silva RA, Hurley JJ, Arron BL, Sarfraz N, Lee HN, Ghali GE, Gamble JW (2021). Economic impact of COVID-19 pandemic on healthcare facilities and systems: international perspectives. Best Pract Res Clin Anaesthesiol.

[24] García-Fernández L, Romero-Ferreiro V, López-Roldán PD, Padilla S, Calero-Sierra I, Monzó-García M, Pérez-Martín J, Rodriguez-Jimenez R: Mental health impact of COVID-19 pandemic on Spanish healthcare workers. Psychol Med 2020:1–3. 10.1017/S0033291720002019 PMC7272696 32456735

[25] Rana W, Mukhtar S, Mukhtar S (2020). Mental health of medical workers in Pakistan during the pandemic COVID-19 outbreak. Asian J Psychiatr.

[26] Koh D, Lim MK, Chia SE, Ko SM, Qian F, Ng V, Tan BH, Wong KS, Chew WM, Tang HK (2005). Risk Perception and impact of severe acute respiratory syndrome (SARS) on work and personal lives of healthcare workers in Singapore what can we learn?. Med Care.

[27] McMahon SA, Ho LS, Brown H, Miller L, Ansumana R, Kennedy CE (2016). Healthcare providers on the frontlines: a qualitative investigation of the social and emotional impact of delivering health services during Sierra Leone's Ebola epidemic. Health Policy Plan.

[28] Mediavilla R, Fernández-Jiménez E, Andreo J, Morán-Sánchez I, Muñoz-Sanjosé A, Moreno-Küstner B, Mascayano F, Ayuso-Mateos JL, Bravo-Ortiz M-F, Martínez-Alés G: Association between perceived discrimination and mental health outcomes among health workers during the initial COVID-19 outbreak. Revista de Psiquiatría y Salud Mental 2021. 10.1016/j.rpsm.2021.06.001 PMC8253602 34153496

[29] Singh R, Subedi M (2020). COVID-19 and stigma: Social discrimination towards frontline healthcare providers and COVID-19 recovered patients in Nepal. Asian J Psychiatr.

[30] Kuriyama A, Shikino K, Moriya M, Sadohara M, Nonaka S, Nagasaki K, Nishimura Y, Matsuo T, Muramatsu K, Makiishi T (2022). Burnout, depression, anxiety, and insomnia of internists and primary care physicians during the COVID-19 pandemic in Japan: a cross-sectional survey. Asian J Psychiatr.

[31] Williams OD (2020). COVID-19 and Private health: market and governance failure. Development (Rome).

[32] Association ID, Corporation IF: Impacts of COVID-19 on the Private Sector in Fragile and Conflict-Affected Situations. EMCompass 2020(Note 93).

[33] Lau J, Tan DH, Wong GJ, Lew YJ, Chua YX, Low LL, Koh GC, Kwek TS, Toh SE, Tan KK (2021). The impact of COVID-19 on private and public primary care physicians: a cross-sectional study. J Infect Public Health.

[34] Ahmed F, Sifat RI (2021). The impact of the COVID-19 pandemic on the mental health of the rickshaw-puller in Bangladesh. J Loss Trauma.

[35] Murphy M, Scott LJ, Salisbury C, Turner A, Scott A, Denholm R, Lewis R, Iyer G, Macleod J, Horwood J (2021). Implementation of remote consulting in UK primary care following the COVID-19 pandemic: a mixed-methods longitudinal study. Br J Gen Pract.

[36] Andrew A, Cattan S, Costa-Dias M, Farquharson C, Kraftman L, Krutikova S, Phimister A, Sevilla A: Family time use and home learning during the COVID-19 lockdown. In. London: The Institute for Fiscal Studies; 2020: 70. 10.1111/1475-5890.12240 PMC7753283 33362314

[37] Gash V, Mertens A, Gordo LR (2012). The influence of changing hours of work on women's life satisfaction*. Manch Sch.

[38] Yerkes MA, André SCH, Besamusca JW, Kruyen PM, Remery C, van der Zwan R, Beckers DGJ, Geurts SAE (2020). 'Intelligent' lockdown, intelligent effects? Results from a survey on gender (in)equality in paid work, the division of childcare and household work, and quality of life among parents in the Netherlands during the Covid-19 lockdown. PLoS ONE.

[39] Möhring K, Naumann E, Reifenscheid M, Wenz A, Rettig T, Krieger U, Friedel S, Finkel M, Cornesse C, Blom AG (2021). The COVID-19 pandemic and subjective well-being: longitudinal evidence on satisfaction with work and family. Eur Soc.

[40] Benzeval M, Burton J, Crossley TF, Fisher P, JÃ¤ckle A, Perelli-Harris B, Walzenbach S: Briefing note COVID-19 survey: family relationships. In: Understanding Society Working Paper. Colchester; 2020.

[41] Kang L, Li Y, Hu S, Chen M, Yang C, Yang BX, Wang Y, Hu J, Lai J, Ma X (2020). The mental health of medical workers in Wuhan, China dealing with the 2019 novel coronavirus. Lancet Psychiatry.

