# Supplementary material for: Psychological health and wellbeing of primary healthcare workers during COVID-19 pandemic in Malaysia: a longitudinal qualitative study
Source: BMC Prim Care. 2022 Oct 13;23:261. doi: 10.1186/s12875-022-01870-0 (PMC9558025; doi:10.1186/s12875-022-01870-0)
Supplement: Supplementary file 1 — Additional file 1: [file 12875_2022_1870_MOESM1_ESM.docx]

**Appendix A**

**Topic Guide**

**First Interview:**

1. Can you describe your job?
2. Are you actively working as a frontliner during the COVID-19 pandemic?
3. If no, why?
4. If yes, how are you involved in patient management during the COVID-19 outbreak?

Prompt: job scope, risk level, patient load, types of patients

1. How has the pandemic affected your job or work?
2. Can you share with us your experience as a HCW in primary care during this pandemic?
   1. What do you think about the pandemic?

Prompt: How susceptible do you think you are to COVID-19? What did you do to reduce the risk?

- 1. What do you feel about the pandemic?
  2. Has this pandemic affected your behavior?
     1. At home? If yes, how? If not, why not?

Prompt: preventive measures? Daily home routine? Change of role at home? Child care? Child schooling? House work?

- - 1. At work? If yes, how? If not, why not?

Prompt: Working routine? PPE? Preventive measures?

- - 1. Socially

1. What supports you during this time?
   1. At home?
      1. Prompt: Can you share with us the support and resources available to you during this pandemic? Support from spouse? Family support?
      2. Prompt: What additional resources, information and support do you think would be beneficial?
   2. At work?
      1. Prompt: Can you share with us the support and resources available to you during this pandemic?
      2. Prompt: What additional resources, information and support do you think would be beneficial?

1. Can you give me an example of events that caused panic or were frightening at work during this time?

How did you handle the situations? How did you cope: able to manage thoughts, emotions and behaviors? What resources or support had helped? What else could have helped?

1. How has the pandemic affected you and your close ones?

Prompt: How susceptible do you think you are to COVID-19? Your family? Others? What did you do to reduce the risk?

1. What do you think is going to happen in the future?

Prompt: How long do you think the COVID-19 pandemic is going to be? What is your view of the future? How do you think you will be affected in the long run?? How bad do you think COVID-19 is?

1. Are there any other issues to bring up related to you as a HCW in primary care during this COVID-19 pandemic?

**Second interview:**

1. How has the pandemic affected your job or work (since we last spoke)?
2. What new challenges or events had occurred? How did you cope?
3. What skills or knowledge would have been helpful to help you cope with these challenges?
4. What do you think is going to happen in the future?

Prompt: How long do you think the COVID-19 pandemic is going to be? What is your view of the future? How do you think you will be affected in the long run?? How bad do you think COVID-19 is?
